# Zebrafish as a Vertebrate Model for Studying Nodavirus Infections

**DOI:** 10.3389/fimmu.2022.863096

**Published:** 2022-03-24

**Authors:** Raquel Lama, Patricia Pereiro, Antonio Figueras, Beatriz Novoa

**Affiliations:** Instituto de Investigaciones Marinas (IIM), Consejo Superior de Investigaciones Científicas (CSIC), Vigo, Spain

**Keywords:** nodavirus, viral encephalopathy and retinopathy (VER), zebrafish, immune response, RNA-Seq

## Abstract

Nervous necrosis virus (NNV) is a neurotropic pathogenic virus affecting a multitude of marine and freshwater fish species that has a high economic impact on aquaculture farms worldwide. Therefore, the development of new tools and strategies aimed at reducing the mortality caused by this virus is a pivotal need. Although zebrafish is not considered a natural host for NNV, the numerous experimental advantages of this species make zebrafish an attractive model for studying different aspects of the disease caused by NNV, viral encephalopathy and retinopathy (VER). In this work, we established the best way and age to infect zebrafish larvae with NNV, obtaining significant mortalities in 3-day-postfertilization larvae when the virus was inoculated directly into the brain or by intramuscular microinjection. As occurs in naturally susceptible fish species, we confirmed that after intramuscular injection the virus was able to migrate to the central nervous system (CNS). As expected, due to the severe damage that this virus causes to the CNS, alterations in the swimming behavior of the zebrafish larvae were also observed. Taking advantage of the existence of transgenic fluorescent zebrafish lines, we were able to track the migration of different innate immune cells, mainly neutrophils, to the site of infection with NNV *via* the brain. However, we did not observe colocalization between the viral particles and neutrophils. RNA-Seq analysis of NNV-infected and uninfected larvae at 1, 3 and 5 days postinfection (dpi) revealed a powerful modulation of the antiviral immune response, especially at 5 dpi. We found that this response was dominated by, though not restricted to, the type I interferon system, the major defence mechanism in the innate immune response against viral pathogens. Therefore, as zebrafish larvae are able to develop the main characteristic of NNV infection and respond with an efficient immune arsenal, we confirmed the suitability of zebrafish larvae for modelling VER disease and studying different aspects of NNV pathogenesis, immune response and screening of antiviral drugs.

## 1 Introduction

Zebrafish (*Danio rerio*) are a very versatile animal model widely used in the study of a multitude of processes and disciplines. Among them, zebrafish are an excellent tool for understanding host–pathogen interactions during the course of infectious diseases (1, 2). Zebrafish possess innate and adaptive immunity resembling that of mammals and other higher vertebrates. However, in the early stages, the larvae rely exclusively on their innate immune system, as the cells responsible for the adaptive responses are not functionally mature until 4 or 6 weeks postfertilization (3). This fact makes zebrafish larvae a very attractive model for studying the first line of defence against a pathogen, such as the action of primary immune cells, macrophages (4) and neutrophils (5) and the role of the main cytokines involved in the immune response, without interference from the adaptive response. In addition, other advantages of using early stages of zebrafish larvae are the large numbers of offspring, a short generation time, tolerance to anaesthesia, a small body size, and transparency, which allows easy visualization of the whole body by live imaging (6–8). At the genetic level, the zebrafish genome sequence has been extensively revised and refined, enabling the rapid accumulation of loss- or gain-of-function mutants and the generation of transgenic lines that allow traceability of different cell types.

Although no viruses, with some exceptions, are typically known to naturally infect zebrafish and cause massive mortality episodes (9, 10), several viruses (including those infecting humans) have been studied using zebrafish as a model of infection. In the case of fish, the main diseases caused by viruses have been reproduced in zebrafish: rhabdoviruses such as spring viremia of carp virus (SVCV) (11–15), snakehead rhabdovirus (SHRV) (16), viral haemorrhagic septicemia virus (VHSV) (17), and infectious haematopoietic necrosis virus (IHNV) (7); birnaviruses such as infectious pancreatic necrosis virus (IPNV) (18, 19); iridoviruses such as infectious spleen and kidney necrosis virus (ISKNV) (20, 21) or European sheatfish virus (ESV) (22); and nodaviruses such as nervous necrosis virus (NNV) (23, 24). Interestingly, two publications reported that zebrafish can be naturally infected by different nodaviruses (25, 26); this can probably occur as a consequence of increases in temperature and crowding (27). In both cases, extensive vacuolations were seen in the brain and retina (25, 26), and erratic swimming behavior and mortality episodes were reported by Binesh (25).

Viral encephalopathy and retinopathy (VER) disease, caused by NNV, is one of the most devastating diseases affecting commercial fish species around the world, such as European sea bass (*Dicentrarchus labrax*), Atlantic cod (*Gadus morhua*) or grouper (*Epinephelus* spp.), among others. This icosahedral naked positive-sense single-stranded RNA virus has a neurotropic nature and replicates in the nervous system (e.g., brain, retina and spinal cord) (28), causing a very characteristic abnormal and erratic swimming behavior in susceptible fish species, accompanied by other less specific signs (exophthalmia, swim bladder hyperinflation, skin darkening, anorexia or lethargy) (29). Despite the relevance of this disease and the high economic impact caused by NNV and although many studies have been conducted in commercial species to analyse different aspects of NNV infection (30–39), few studies have leveraged the benefits of zebrafish to investigate NNV infection. Furusawa et al. (40) tried to establish experimental infections in adult zebrafish but without success. Later, Lu et al. (23) were able to reproduce NNV infection in both adult zebrafish and larvae, and relevant mortalities were observed in larvae microinjected with the virus, which was subsequently confirmed by Morick et al. (24) after bath exposure. The susceptibility of zebrafish larvae to NNV creates an opportunity to easily screen anti-NNV compounds in an *in vivo* model, as was performed with the antiviral drug ribavirin (41). Although the immune response to NNV in this fish species seems to indicate the relevance of the type I interferon (IFN) system (23, 42–44), a complete transcriptome response to NNV has not been previously determined in zebrafish.

In this work, we sought to improve the knowledge of VER disease through the use of zebrafish larvae as an NNV infection model, leveraging different imaging methods and transgenic fish lines, and by analysing their transcriptome response after challenge with the virus. We confirmed that zebrafish larvae are susceptible to NNV when they are challenged at 3 days postfertilization (dpf), especially when infections are conducted *via* the brain or intramuscularly. Indeed, NNV particles were detected by immunofluorescence in the heads of larvae infected by intramuscular injection, confirming the migration of the virus to nervous tissues. Moreover, as occurs in commercial fish species susceptible to the virus, the infection altered the swimming behavior of the larvae, reflecting their suitability as a good model for studying different aspects of the infection. An efficient immune response against NNV is mounted in the larvae, with a significant migration of neutrophils to the brain, although these cells were not found to colocalize with the virus. At the transcriptome level, a time-increasing immune response is mounted against the virus, which is mainly characterized by a large overexpression of those genes belonging to the type I IFN system but also with a vast representation of other immune processes.

## 2 Materials And Methods

### 2.1 Fish

The embryos and larvae used in this study were obtained from our experimental facilities, where the animals were cultured using established protocols (45, 46). Different fish lines were used: wild-type (WT) zebrafish, AB wild-type line (AB), Tübingen wild-type line (TU), and the transgenic lines Tg(*mpx*:GFP), Tg(*mpeg*:mCherry) and Tg(*lyz*:DsRed2), with neutrophils, macrophages and lysozyme-expressing cells, respectively. The eggs were obtained according to protocols described in The Zebrafish Book (45) and maintained at 28°C in E3 egg water (5 mM NaCl, 0.17 mM KCl, 0.33 mM CaCl_2_, 0.33 mM MgSO_4_, and 0.00005% methylene blue). All experimental procedures were reviewed and approved by the CSIC National Committee of Bioethics under approval number ES3605702020012020/13/FUN.01/INM06/BNG.

### 2.2 Virus

The nodavirus red-spotted grouper nervous necrosis virus (RGNNV) (strain 475-9/99) was kindly provided by the Institute Zooprofilattico delle Venezie (Italy) after isolation from diseased sea bass (47). The virus was propagated in the snakehead-fish cell line SSN-1 (ECACC 96082808) cultured in L-15 medium (Gibco) supplemented with 2 mM L-glutamine (Gibco), 2% FBS (Gibco), and 1% penicillin/streptomycin solution (Invitrogen) and incubated at 25°C. The viral stock was titrated into 96-well plates using the Reed-Müench method (48), and aliquots were stored at −80°C until use.

### 2.3 Mortality Assays in Zebrafish Larvae Infected With NNV

**Figure 1:**
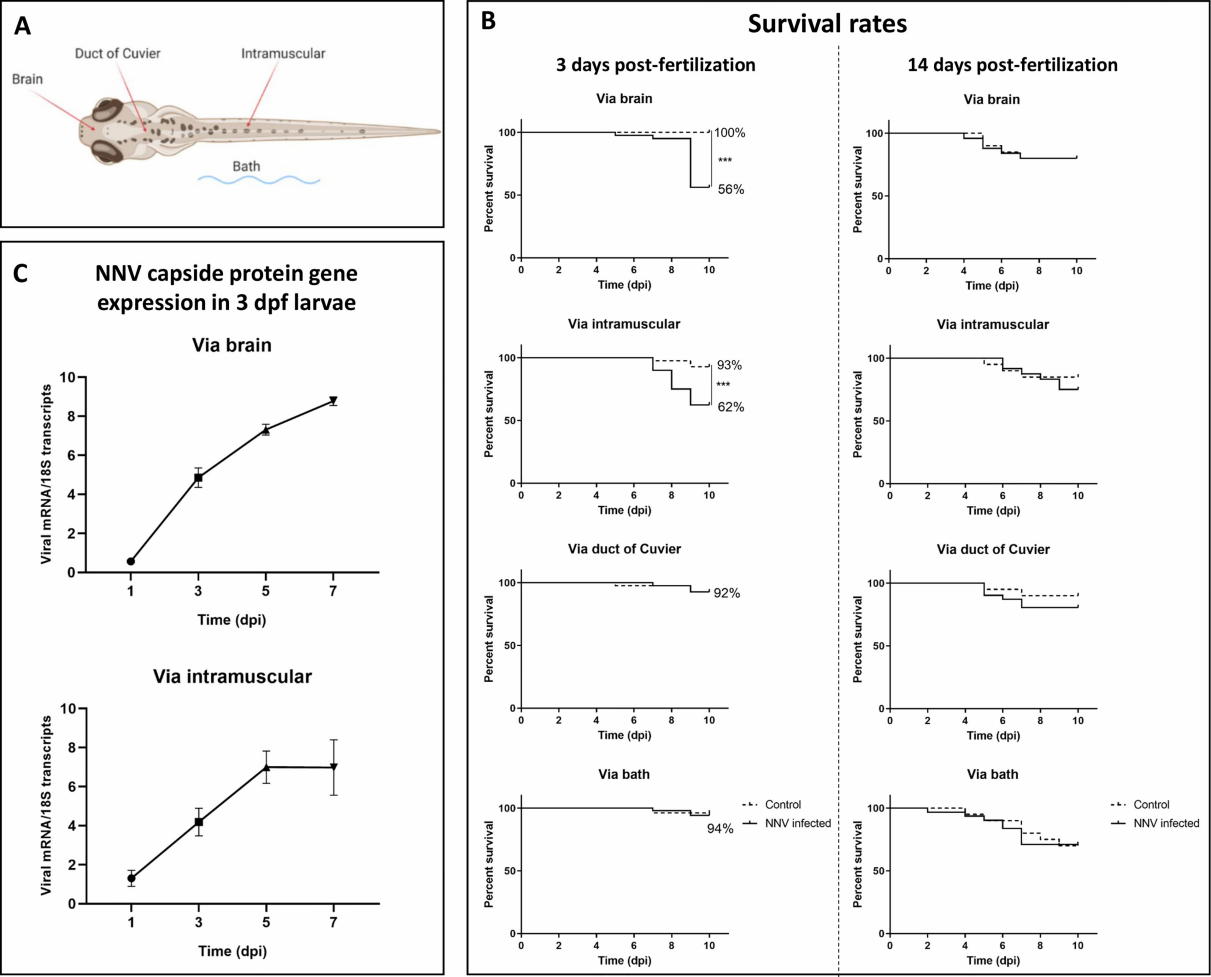
Survival rates of zebrafish larvae challenged with NNV through different infection routes and NNV replication. **(A)** Schematic representation of the different infection routes used to determine the susceptibility of zebrafish larvae to NNV. **(B)** Kaplan–Meier survival curves of NNV-infected and uninfected larvae in 3- and 14-dpf larvae. Mortality was registered during the next 10 dpi. **(C)** Quantification of NNV capsid protein gene expression in 3-dpf larvae infected *via* the brain or intramuscularly at different sampling points through qPCR; data are presented as the mean ± SEM of biological replicates. Statistically significant differences are displayed as follows: ***, 0.0001 > p value > 0.001.

To determine the most efficient route and age of infection with NNV, WT zebrafish larvae were infected at 3 and 14 dpf through 4 different routes: a) *via* the brain by microinjecting the virus directly into the area of the head between the eyes to reach the brain, b) *via* the duct of Cuvier (DC) to produce a systemic infection, c) by intramuscular (IM) injection by microinjecting into the muscle in the middle of the back, and d) by bath by immersing the larvae in a viral suspension (**Figure 1A**).

Larvae were anaesthetized in zebrafish water containing 160 µg/mL MS-222 (Sigma–Aldrich), placed on an agarose plate and individually microinjected with a glass microneedle using a Narishige MN-151 micromanipulator and a FemtoJet 4x microinjector (Eppendorf). NNV was diluted at an appropriate concentration (10^6^ TCDI_50_/mL) in L15 medium with 0.1% phenol red (a coloured marker to easily visualize the correct injection of the solution into the larvae) just before microinjection of 2 nL of viral suspension to the larvae or 2 nL of L15+0.1% phenol red to the control larvae. Larvae were infected through microinjection into the brain or DC or intramuscularly and by immersion in water containing 10^6^ TCDI_50_/mL NNV. Then, larvae were maintained in Petri dishes at 28°C on a 12 h light-dark cycle. Mortality was recorded daily during the next 10 days in the three biological replicates (10 larvae/replicate) obtained for each condition. The experiments were repeated three times. Additionally, to confirm the mortality caused by the microinjection of the virus *via* the brain, AB and TU larvae (3 dpf) were also infected.

### 2.4 Evaluation of NNV Replication and Gene Expression of Immune Cell Markers in Zebrafish Larvae by Quantitative PCR (qPCR)

To evaluate the progress of NNV infection, larvae (3 dpf) were infected *via* the brain or intramuscularly, and samples were collected at 1, 3, 5 and 7 days postinfection (dpi). Whole larvae were harvested under RNAse-free conditions in pools of 4 larvae each (3 biological replicates/4 larvae per replicate). Total RNA was isolated using the Maxwell^®^ RSC simplyRNA Tissue kit (Promega) in accordance with the manufacturer’s instructions. cDNA synthesis was performed with the NZY First-Strand cDNA Synthesis Kit (NZYtech) using 0.2 µg of total RNA. The qPCRs were performed using specific primers designed with Primer 3 software (49), and their efficiencies were previously tested according to the protocol described by Pfaffl (50). Individual qPCRs were conducted in 25-µl reaction volumes using 12.5 µL of SYBR GREEN PCR Master Mix (Applied Biosystems), 10.5 µL of ultrapure water (Sigma–Aldrich), 0.5 µL of each specific primer (10 µM) and 1 µL of cDNA template. All reactions were performed using technical triplicates in a 7300 Real-Time PCR System thermocycler (Applied Biosystems) with an initial denaturation step (95°C, 10 min), followed by 40 cycles of a denaturation step (95°C, 15 s) and one hybridization-elongation step (60°C, 1 min). Viral replication was detected by the relative gene expression of the NNV capsid protein gene (RNA2) (51). For larvae microinjected in the brain, the gene expression of the neutrophil marker *myeloperoxidase* (*mpx*) and the macrophage marker *macrophage receptor with a collagenous domain* (*marco*) was also evaluated; *mpx* and *marco* primers were previously confirmed to specifically amplify the myeloid zebrafish population (52). The relative expression levels of the different genes were normalized using the Pfaffl method (50) and *18S ribosomal RNA* (*18S*) as a reference gene. The primers used are listed in **Supplementary Table S1**.

### 2.5 Fluorescence Microscopy Images

Whole-mount immunofluorescence assays in zebrafish larvae were performed as follows. WT larvae (3 dpf) were infected *via* IM injection for 2, 6, 24 and 48 h. Then, larvae were fixed overnight (O/N) at 4°C in 4% paraformaldehyde (PFA) diluted in phosphate-buffered saline containing 0.1% Tween-20 (PBST). Larvae were washed twice in PBST, dehydrated in a graded series of methanol/PBST solutions (25% for 5 min, 50% for 10 min and 75% for 5 min), and stored in 100% methanol O/N at -20°C. For immunofluorescence processing, larvae were rehydrated in a graded series of methanol/PBST solutions (75% for 5 min, 50% for 10 min and 25% for 5 min) and washed 4 times for 5 min with PBST. Larvae were bleached by incubating them in bleaching solution (0.8 mL of KOH 10%, 0.3 mL of H_2_O_2_ 30%, 0.1 mL of Tween-80 and 8.8 mL of distilled water) for 5 min, and then they were washed twice for 5 min in PBST. Permeabilization was achieved by incubation with proteinase K at 10 µg/mL. After 2 h at 37°C, larvae were washed twice in PBST and incubated in 2% Tween-20 in PBS for 24 h at room temperature (RT). Larvae were washed and blocked in 1% Tween-20/PBS and 10% lamb serum O/N at RT. Anti-sea bass encephalitis virus (anti-DIEV, 1:4.000) (53) was diluted in antibody solution (0.2% Tween-20/PBS and 10% lamb serum) and incubated with the larvae for 3 days at 4°C. Larvae were washed O/N and then incubated for 2 days with Alexa Fluor 546 goat anti-rabbit secondary antibody (Invitrogen) diluted in antibody solution (1:500). After this, larvae were washed O/N in PBST and stained with DAPI for 1 h at RT. After 3 washes with PBS, larvae were stored at 4°C until microscopy examination. Confocal images of fixed larvae were taken using a TSC SPE confocal microscope (Leica). The images were processed using the LAS-AF (Leica Application Suite Advanced Fluorescence) program. The same procedure was conducted for Tg(*mpx*:GFP) larvae (3 dpf) infected *via* the brain with NNV for 24 h. For the WT larvae, samples were also taken at the four sampling points for RNA isolation and qPCR analysis of NNV replication (5 samples of 5 larvae/sample).

Tg(*mpx*:GFP), Tg(*mpeg*:mCherry) and Tg(*lyz*:DsRed2) transgenic larvae (3 dpf) were infected by 4 different routes as explained in Section 2.3, and after 1, 2 and 3 dpi, images of whole larvae were taken using a Nikon AZ100 fluorescence microscope. Larvae were anaesthetized with a 0.01% MS-222 solution. The different immune cells labelled in the transgenic lines were counted using a macro of ImageJ (54) to calculate the percentage of cells that migrated to the brain during infection.

### 2.6 Video Recording of the Swimming Behavior

To analyse the swimming behavior of the fish larvae, two Petri dishes of 10 larvae each, inoculated by the same route (via brain or IM), one containing infected larvae and the other uninfected control larvae, were placed in the same plane of a video recording for 2 consecutive minutes. Recordings of 3- and 14-dpf larvae were made at 3, 6 and 10 dpi with a Leica camera of 48 Mpx, and the images were processed with Photoshop and ImageJ (54) using the Chemotaxis and Migration Tool plugin. The data obtained allowed us to reconstruct the larval movements to calculate the velocity, the accumulated and Euclidean distances and the directionality, parameters that were used to compare the swimming behavior between infected and uninfected control larvae.

### 2.7 High-Throughput Transcriptome Sequencing

To analyse the transcriptome response to NNV in zebrafish larvae infected *via* the brain, the samples collected in Section 2.4 and corresponding to sampling points 1, 3 and 5 dpi were used for high-throughput transcriptome sequencing. The RNA concentration and purity were measured with a Nanodrop ND-1000 spectrophotometer (Nanodrop Technologies Inc., USA), and RNA integrity was analysed in an Agilent 2100 Bioanalyzer (Agilent Technologies Inc., USA) according to the manufacturer’s instructions. All samples showed an RNA integrity number (RIN) over 8.0 and were used for Illumina library preparation. Double-stranded cDNA libraries were constructed using the TruSeq Stranded mRNA Kit Sample Prep Kit (Illumina, USA), and sequencing was performed using Illumina NovaSeq 6000 technology at Macrogen Inc., Korea (Republic of Korea). The raw read sequences were deposited in the Sequence Read Archive (SRA) (http://www.ncbi.nlm.nih.gov/sra) under the BioProject accession number PRJNA799765.

### 2.8 Raw Data Cleaning, Mapping, RNA-Seq and Differential Expression Analysis

CLC Genomics Workbench v. 20.0.4 (CLC Bio, Denmark) was used to filter and trim reads, map the high-quality reads against the last version of the zebrafish genome (GRCz11) and perform the differential expression analyses. Raw reads were trimmed to remove the adaptor sequences and low-quality reads. RNA-Seq analyses were conducted with the following parameters: length fraction = 0.8, similarity fraction = 0.8, mismatch cost = 2, insertion cost = 3 and deletion cost = 3. The expression values were set as transcripts per million (TPM). Finally, a differential expression analysis test was used to compare gene expression levels and to identify differentially expressed genes (DEGs). Transcripts with fold change (FC) values > |2| and false discovery rate (FDR) values < 0.05 were retained for further analyses. To identify and quantify the directions of variability in the data, a principal component analysis (PCA) plot was constructed using the original expression values. Using the TPM values of the selected DEGs, heatmaps for each sampling point were constructed using the complete linkage method with Euclidean distance. Both PCA and heatmaps were constructed using the web tool Clustvis (55) (https://biit.cs.ut.ee/clustvis/), and a Venn diagram was constructed with the InteractiVenn web tool (56) (http://www.interactivenn.net/).

### 2.9 Gene Ontology (GO) Enrichment and Kyoto Encyclopedia of Genes and Genomes (KEGG) Pathway Analysis

For the DEGs between NNV-infected and noninfected zebrafish larvae, we conducted a GO enrichment analysis of biological processes and a KEGG pathway analysis using the functional annotation tool DAVID v. 6.8 (57, 58) (https://david.ncifcrf.gov/summary.jsp). For the GO and KEGG analyses, a p value < 0.05 was employed.

### 2.10 qPCR Validation of RNA-Seq Data

The RNA-Seq results were validated by qPCR analysis of five immune genes significantly modulated by NNV infection (*ifnphi1*, *il1b*, *mxe*, *tnfa* and *marco*), as mentioned above for NNV detection (Section 2.4). The primers used are listed in **Supplementary Table S1**. The correlation between the fold changes obtained by RNA-Seq and qPCR was calculated using Pearson’s correlation coefficient.

### 2.11 Statistical Analysis

Kaplan–Meier survival curves were analysed with a log-rank (Mantel–Cox) test. For the remaining experiments, the results were represented graphically as the mean ± standard error of the mean (SEM), and significant differences were obtained using Student’s t test and displayed as **** (< 0.0001, *** (0.0001 < p < 0.001), ** (0.001 < p < 0.01) or * (0.01 < p < 0.05).

## 3 Results

### 3.1 Assessment of Susceptibility to NNV in Zebrafish Larvae Through Different Infection Routes and Larval Developmental Stages

Our results showed that the most effective route of infection with NNV in 3-dpf larvae was *via* the brain, with the survival of the larvae being 56%, whereas their corresponding uninfected controls showed a survival of 100% (**Figure 1B**). Infection *via* IM microinjection also showed significant differences between NN-infected and uninfected larvae, with a survival of 62% for the infected larvae and 93% for the control larvae (**Figure 1B**). Infections *via* DC or by bath did not show significantly different survival rates compared with their corresponding uninfected controls (**Figure 1B**). The lower survival of the 3-dpf WT larvae infected with NNV *via* the brain was also confirmed in the AB and TU zebrafish strains (**Supplementary Figure S1**). However, when the larvae were inoculated under the same conditions but at 14 dpf, the survival rates of the infected larvae were not significantly different from those of the uninfected controls (**Figure 1B**).

As significant mortalities after NNV challenge were observed in 3-dpf larvae infected *via* the brain and intramuscularly, we wanted to assess the replication of the virus in these larvae over time. In larvae inoculated both directly into the brain and through IM microinjection, time-increasing detection of the capsid protein gene was observed; however, in the case of IM microinjection, viral replication seemed to be stable between 5 and 7 dpi (**Figure 1C**).

### 3.2 Kinetic Analysis of Swimming Behavior by a Video Tracking System

Because alterations in swimming behavior are commonly observed in fish naturally susceptible to NNV, we wanted to investigate this fact in 3- and 14-dpf zebrafish larvae infected with NNV *via* the brain or intramuscularly. For this, a video tracking analysis was used to determine if the infection produced changes in their behavioral pattern by analysing certain measurable parameters of the larvae, such as velocity (mm/sec), accumulated distance (mm) of larval path, Euclidean distance (mm) (length of the straight line between the starting point and endpoint of the larvae), and directionality (calculated by comparing the Euclidean distance to the accumulated distance, which represents a measurement of the directness of larval trajectories). Data are represented as the fold change (FC) of infected larvae compared with their uninfected controls (FC = 1, dotted lines).

**Figure 2:**
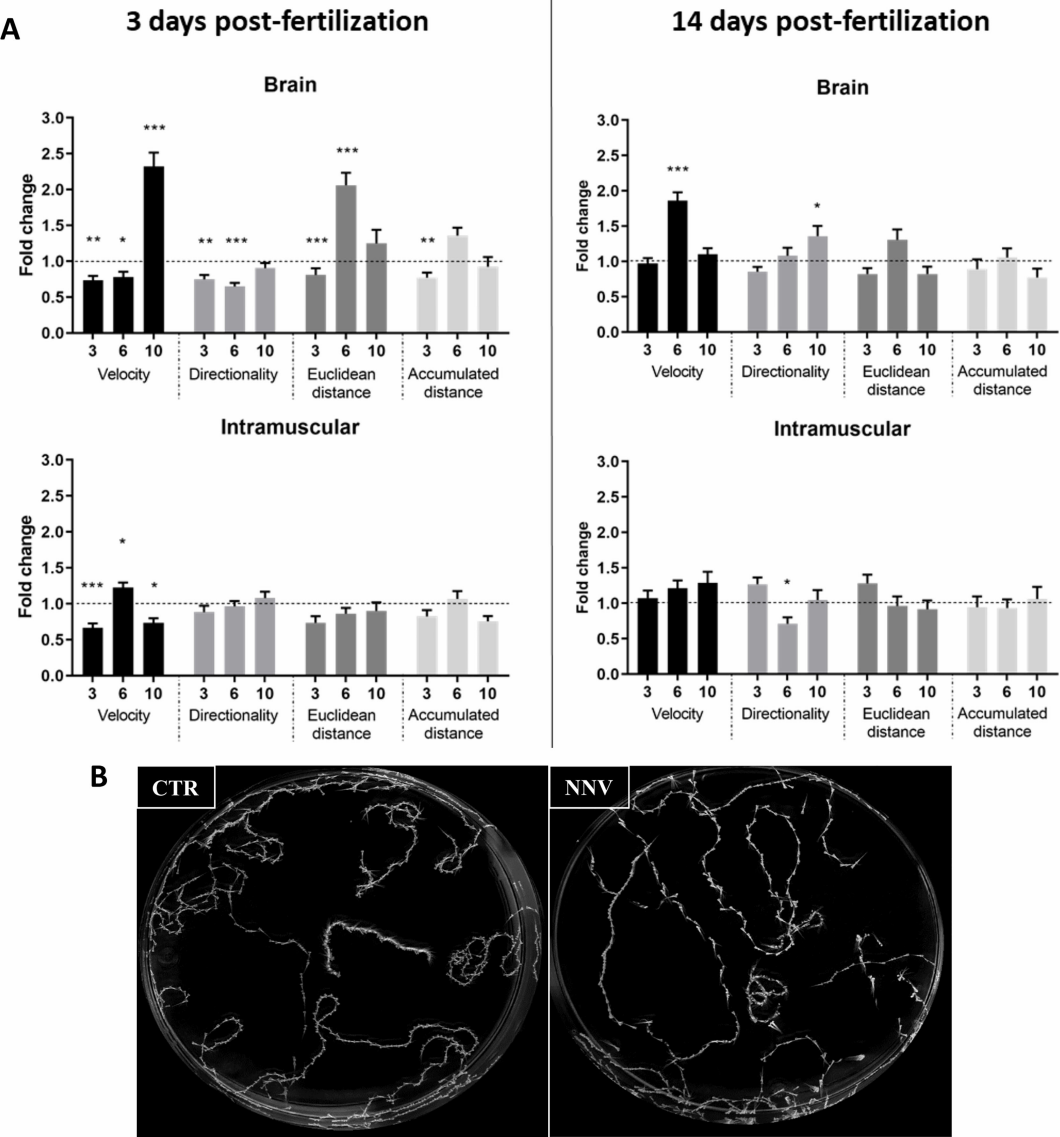
Image analysis of the swimming behavior of zebrafish larvae (3 and 14 dpf) infected with NNV *via* brain or IM. **(A)** Comparison of velocity, directionality, accumulated distance and Euclidean distance parameters between NNV-infected and the corresponding uninfected control larvae. Video tracking of zebrafish larvae was conducted at different times postinfection (3, 6, and 10 dpi). The fold change (FC) of infected larvae compared with their uninfected control (Control FC = 1, dotted lines) was calculated. The graphs represent the mean ± SEM of the biological replicates. Statistically significant differences are displayed as follows: ***, 0.0001 > p value > 0.001; **, 0.001 > p value > 0.01; *, 0.01 > p value > 0.05. **(B)** Example of maximal projection of the video recorded for 3-dpf larvae infected *via* brain and the corresponding controls at 6 dpi.

As shown in **Figure 2A**, in 3-dpf larvae infected *via* the brain, the velocity, directionality and Euclidean distance were found to be significantly different between infected and uninfected larvae at least at one of the analysed times. On the other hand, IM microinjection in 3-dpf larvae produced only significant alterations in velocity. As expected, due to the absence of significantly different mortalities at 14 dpf between infected and uninfected larvae, alterations in these parameters were lower at this age, with only certain significant effects on velocity and directionality in those larvae inoculated *via* the brain and directionality in the larvae inoculated intramuscularly (**Figure 2A**). **Figure 2B** presents an example of the maximum projections used for the analysis of the videos.

### 3.3 Distribution of NNV Virions in Zebrafish Larvae

**Figure 3:**
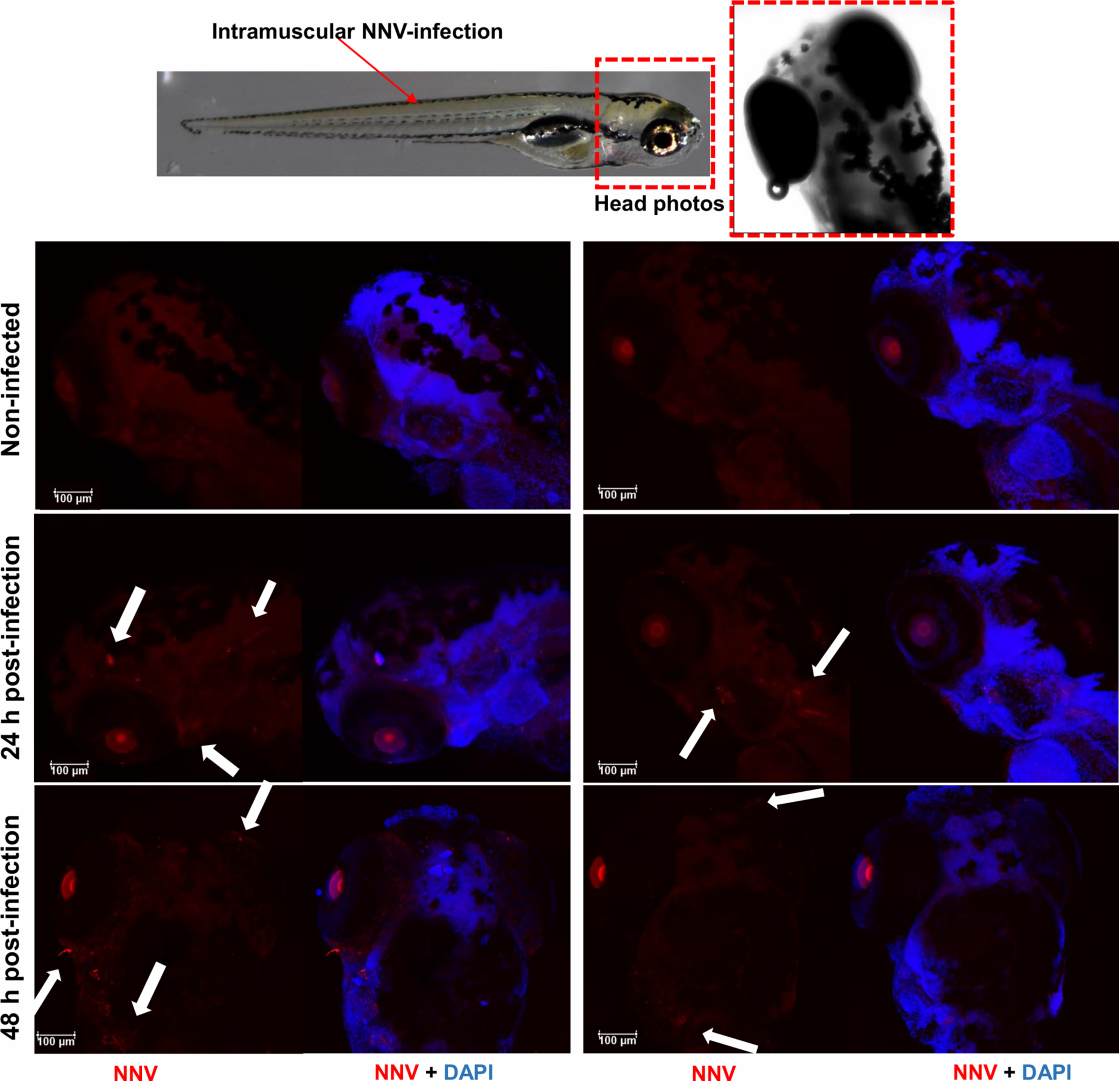
Whole-mount immunofluorescence of zebrafish larvae infected by intramuscular microinjection with NNV. Confocal images of the head from uninfected and NNV-infected larvae at 24 and 48 hpi. NNV particles are stained red, and cell nuclei are stained blue (DAPI). White arrows denote the position of NNV-infected cells.

One of the powerful advantages provided by the use of zebrafish larvae as a working model is the possibility of performing whole-mount immunofluorescence staining, as it allows antigen–antibody interactions to be located without preparing sections of the larvae. Thus, using the anti-DIEV antibody, we were able to locate NNV inside the larvae and confirm that the virus can migrate to the cephalic region after IM infection (**Figure 3**). The virus was not detected until 24 h postinfection (data at 2 and 6 hours postinfection (hpi) not shown), but NNV particles were already observed after 24 h in different locations of the head, and at 48 hpi the virus was also detected in the eye area (**Figure 3**). According to this, the virus was practically undetectable by qPCR at 2 and 6 hpi, whereas a time-increasing detection was observed at 24 and 48 hpi (**Supplementary Figure S2**).

### 3.4 Effects of NNV Challenge on Innate Immune Cells

The existence of transgenic lines in zebrafish provides an easy-to-use working tool that gives us very useful information. To study the immune response of zebrafish to NNV infection, different transgenic lines were used. The transgenic line Tg(*lyz*:DsRed2) was used to detect cells expressing the *lysozyme c* gene (*lyz*) corresponding to myeloid precursors with lysozyme activity. The transgenic line Tg(*mpx*:GFP) was used to detect cells expressing the *myeloperoxidase* gene (*mpx*), a specific marker of differentiated neutrophils. On the other hand, the transgenic line Tg(*mpeg*:mCherry) was used to detect cells expressing *macrophage-expressed gene* (*mpeg*) (macrophages). The larvae were infected by 4 different infection routes, and these cell types were counted at 1, 2 and 3 dpi in the cephalic region. The number of cells that migrated to the head after infection was determined.

**Figure 4:**
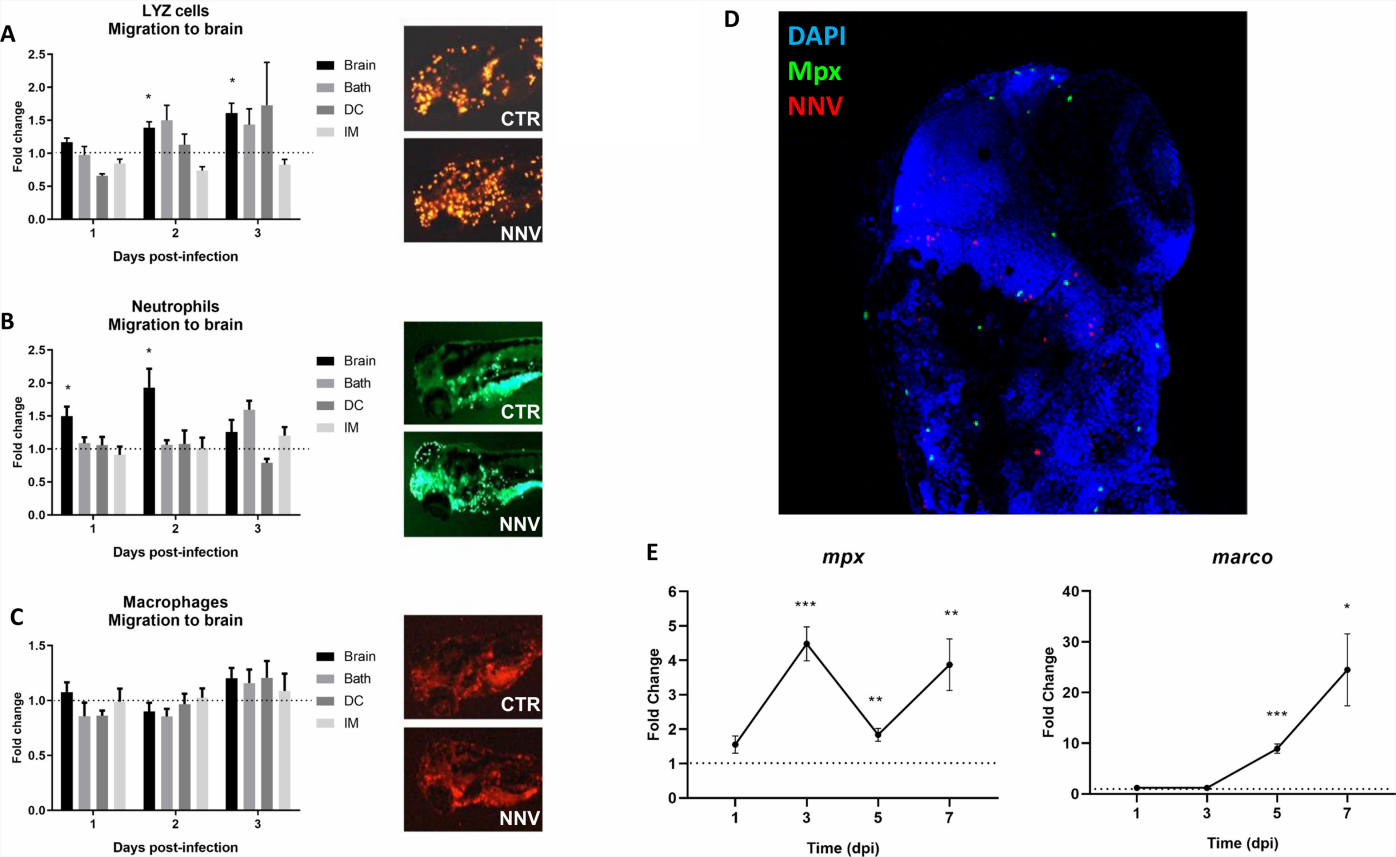
Visualization and analysis of innate immune cell migration to the head of 3 pdf larvae infected through different routes. The transgenic zebrafish lines **(A)** Tg(*lyz*:DsRed2), **(B)** Tg(*mpx*:GFP) and **(C)** Tg(*mpeg*:mCherry) were used to analyse the migration of myeloid precursors with lysozyme activity, neutrophils and macrophages, respectively, to the cephalic region. Larvae were infected through the 4 infection routes analysed in this study, and the cells were counted at 1, 2 and 3 dpi. Fluorescent immune cells were counted using ImageJ, and the graphs represent the difference in fold change of the number of cells located in the head from infected larvae compared to their corresponding uninfected control larvae (control FC = 1 in the dotted line). Representative images of the three transgenic lines at 2 dpi were included. **(D)** Whole-mount immunofluorescence of Tg(*mpx*:GFP) transgenic larvae infected *via* the brain with NNV at 1 dpi; neutrophils are displayed in green, NNV are displayed in red, and nuclei are displayed in blue. No colocalization between NNV particles and neutrophils was observed. **(E)** Expression analysis of marker genes of the two major innate immune cells (*mpx* – neutrophils, *marco* – macrophages) in NNV-infected and uninfected larvae at different times postinfection. Each sample (5 biological replicates, pools of 4 larvae each) was normalized to the *18S* gene. The normalized expression values were standardized against their respective controls (Control FC = 1, dotted lines). For **(A–C, E)**, the graphs represent the mean ± SEM of the biological replicates. Statistically significant differences are displayed as follows: ***, 0.0001 > p value > 0.001; **, 0.001 > p value > 0.01; *, 0.01 > p value > 0.05.

The data represent the differences in the number of cells in infected larvae compared with the uninfected control larvae (control FC = 1 in the dotted line). Lyz+ cells showed significantly higher migration to the head at 48 and 72 hpi when the infection was carried out *via* the brain, whereas no differences were observed for the other infection routes (**Figure 4A**). A similar tendency was observed for neutrophils (Mpx+), which showed significant migration to the head at 24 and 48 hpi in larvae infected *via* the brain, and no effects were observed for the other infections (**Figure 4B**). Although a significant migration of neutrophils to the brain was observed in the larvae inoculated *via* the brain, confocal microscopy analysis showed that these cells did not colocalize with the virus (**Figure 4D**). Finally, no macrophage (Mpeg+) migration to the brain was observed by any route of infection (**Figure 4C**). Interestingly, the expression of the macrophage marker gene *marco* significantly increased after NNV infection in a time-dependent manner, with more marked increases than those observed for the neutrophil marker gene *mpx* (**Figure 4E**).

### 3.5 High-Throughput Sequencing, Mapping Information and PCA Distribution

**Figure 5:**
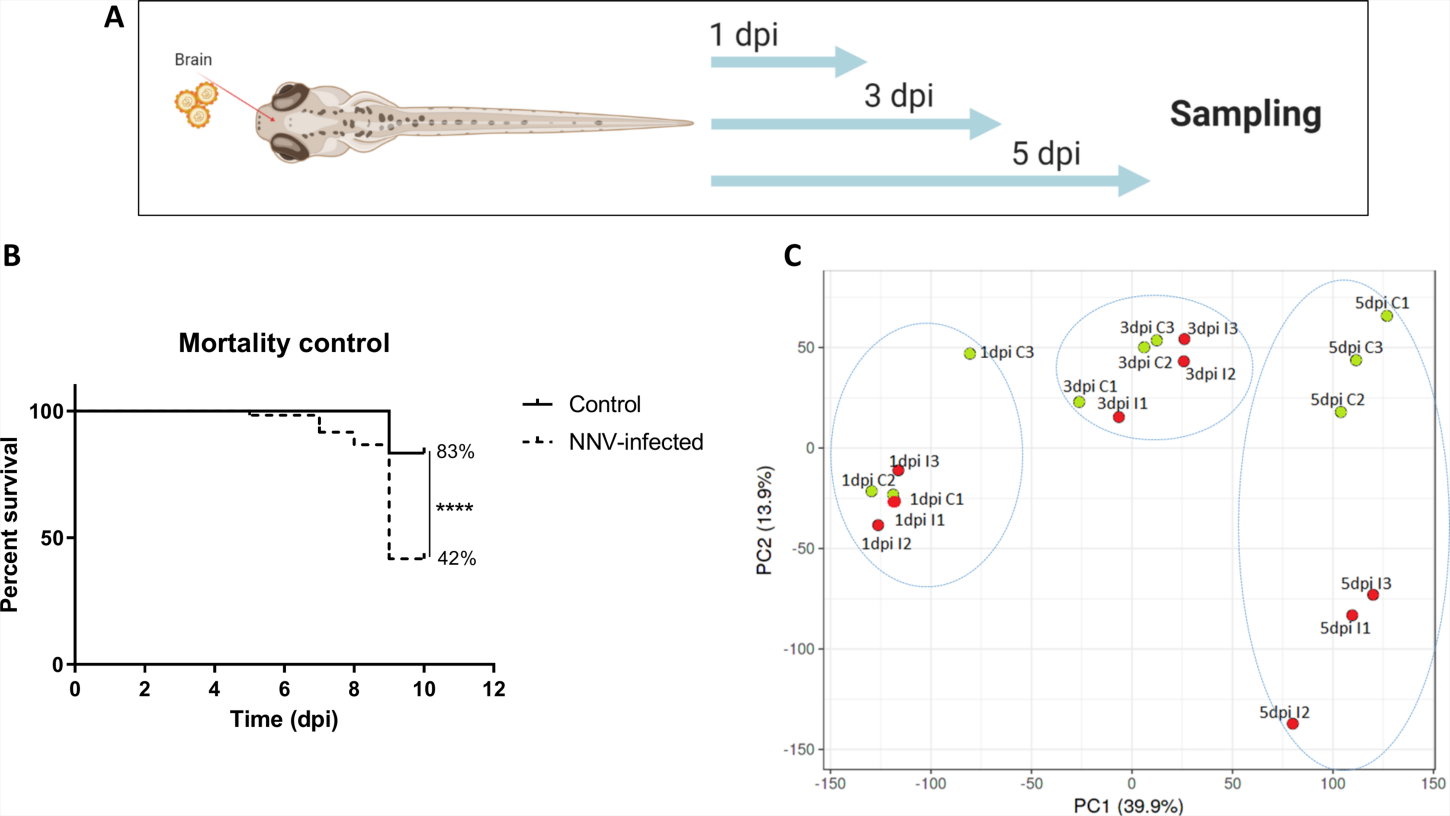
Transcriptome analysis of 3-dpf zebrafish larvae infected with NNV *via* the brain. **(A)** RNA-Seq experimental design: Zebrafish larvae were microinjected *via* the brain, and three pools of infected and uninfected larvae were sampled at 1, 3, and 5 days postinfection for RNA isolation and Illumina sequencing. **(B)** Kaplan–Meier survival curves of NNV-infected and uninfected larvae conducted in parallel to RNA-Seq sampling. Statistically significant differences are displayed as follows: ****, p value < 0.0001. **(C)** Principal component analysis (PCA) of the samples.

To better elucidate the immune response generated in the larvae after challenge with NNV, high-throughput transcriptome sequencing and RNA-Seq analyses were conducted. Samples of 3-dpf larvae infected *via* the brain with NNV or the corresponding control larvae were collected at 1, 3 and 5 dpi for transcriptome sequencing (**Figure 5A**). A parallel assay of mortality was carried out to confirm the success of NNV infection (**Figure 5B**). A total of 541,547,936 reads were obtained from the 18 sequenced samples, with an average value of 30,085,996 reads per sample; of the total raw reads, 99.99% successfully passed the quality control. From these high-quality reads, 97.27% successfully mapped to the zebrafish genome. Therefore, only 2.71% of the reads remained unmapped.

Using the TPM values obtained from RNA-Seq analyses, PCA was performed to determine the sample distribution and to identify the presence of outliers. The PCA plot clearly showed that the NNV-infected samples became more differentiated from the control samples in terms of the overall transcriptome as the postinfection time increased (**Figure 5C**). An evident influence of age on the sample distribution is also observed.

### 3.6 Differentially Expressed Genes, GO Enrichment and KEGG Pathway Analysis

**Figure 6:**
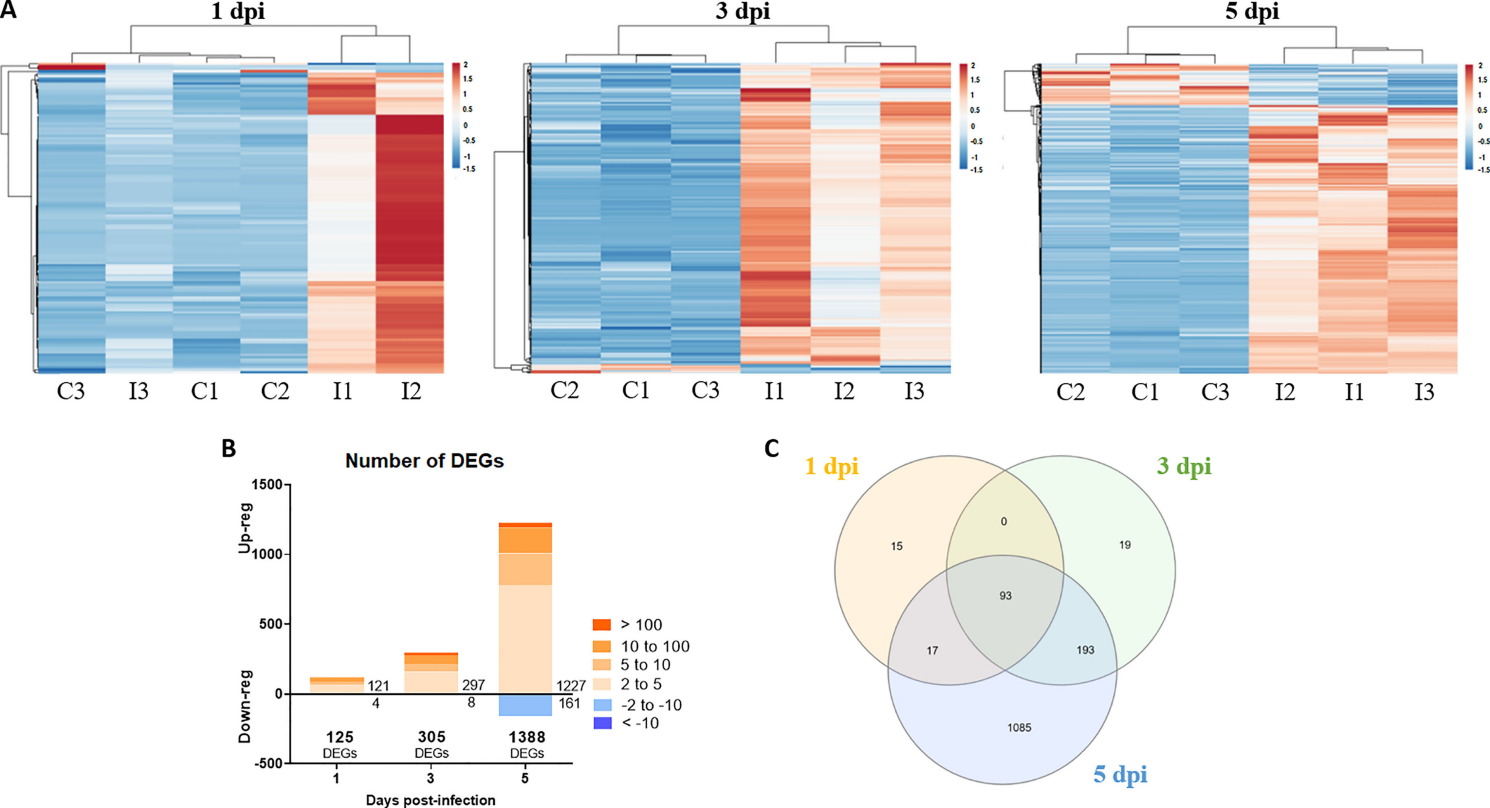
Differentially expressed genes in zebrafish larvae infected with NNV. **(A)** Heatmaps representing the TPM expression values of the DEGs (FC > |2|; FDR < 0.05) modulated at 1, 3 and 5 dpi. Expression levels are represented as row-normalized values on a blue–red colour scale. **(B)** Stacked column chart reflecting the number and intensity (in FC value) of the DEGs identified at the 3 sampling points. **(C)** Venn diagram reflecting the common and exclusive DEGs at each sampling point.

RNA-Seq analyses were conducted to evaluate transcriptome modulation in zebrafish larvae during infection with NNV. Using the obtained data, differentially expressed genes (DEGs) between NNV-infected and uninfected larvae were identified for each sampling point (FC > |2| and FDR value < 0.05) (**Supplementary Files S1**–**S3**). The number of DEGs increased according to the progression of the infection, with 125 DEGs at 1 dpi, 305 DEGs at 3 dpi, and 1,388 DEGs at 5 dpi. The representation of these DEGs in stacked column charts subdividing the number of genes according to the intensity (FC) and direction of regulation (up or down) revealed that most of the genes affected by the infection showed positive regulation (**Figure 6B**). Indeed, the heatmaps representing the TPM values of these DEGs across the different samples also revealed this pattern, with the exception of Replicate 3 from NNV-infected larvae at 1 dpi, whose TMP values of the DEGs at this sampling point were more similar to those observed in the uninfected controls (**Figure 6A**). These differential expression analyses were validated by qPCR amplification of 5 immune genes differentially modulated between NNV-infected and uninfected larvae; a Pearson’s correlation coefficient (*r*) of 0.9755 was obtained for both data groups (**Supplementary Figure S3**).

**Figure 7:**
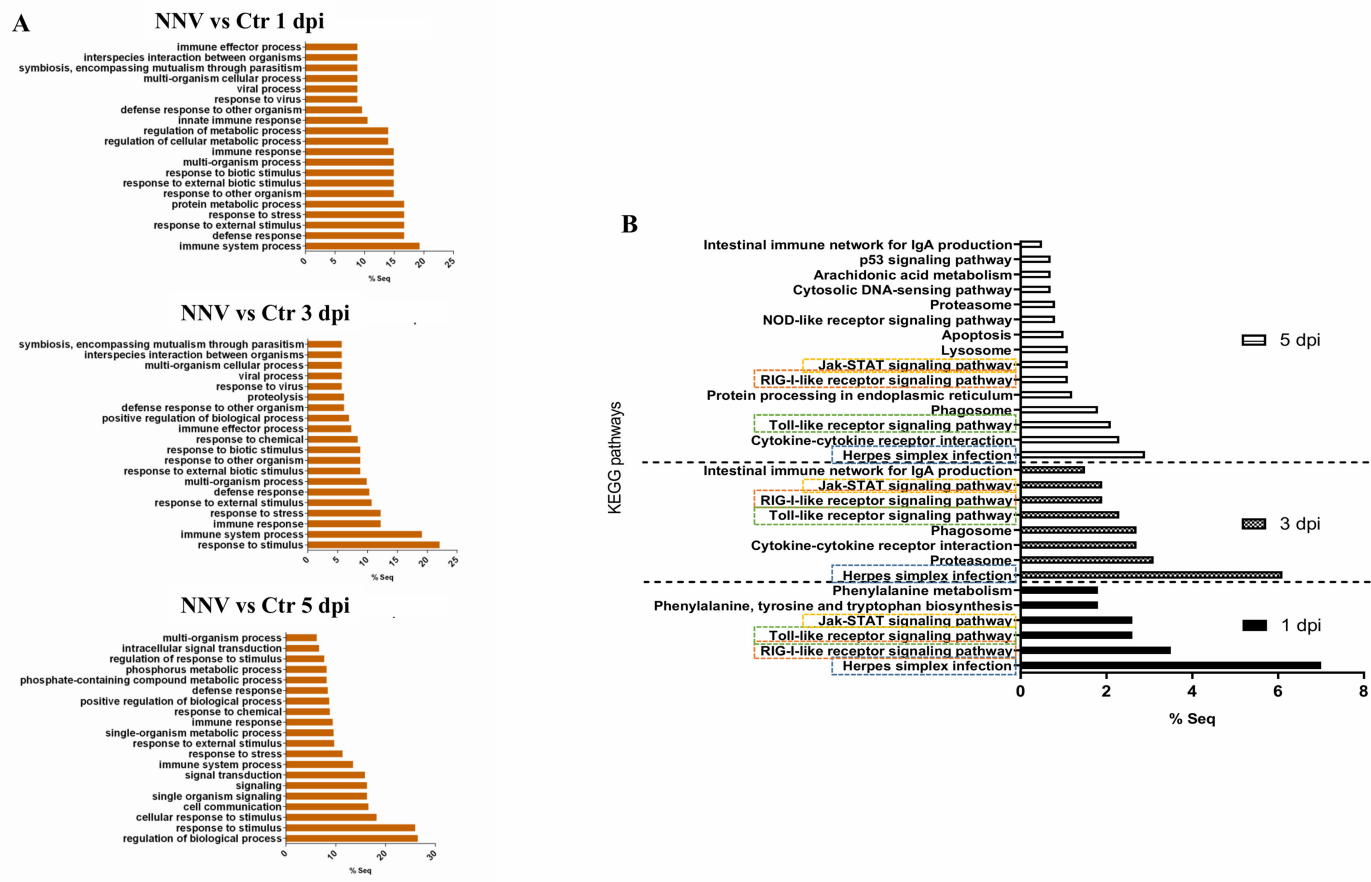
GO terms and KEGG pathways enriched during NNV infection of zebrafish larvae. **(A)** GO biological process terms significantly enriched at 1, 3, and 5 dpi. **(B)** KEGG pathways enriched at 1, 3, and 5 dpi; the four common pathways significantly enriched over time are boxed.

A Venn diagram was constructed to illustrate the number of genes that were commonly regulated along the three sample points (**Figure 6C**); a total of 93 genes were found to be affected at the three times, which corresponded to genes mainly involved in immune response processes. Indeed, when GO enrichment analyses were conducted to explore the biological processes enriched during NNV infection, we observed that more than 60% of the significantly enriched terms were linked to immunity and antiviral response (viral process, response to virus, defence response to other organisms, innate immune response, etc.) (**Figure 7A**). The analysis of the KEGG pathways enriched during NNV infection resulted in a total of six pathways enriched at 1 dpi, eight at 3 dpi, and fifteen at 5 dpi (p value < 0.05) (**Figure 7B**). Four KEGG pathways were modulated throughout the 3 sampling points, and they corresponded to “herpes simplex infection”, “RIG-I-like receptor signalling pathway”, “Toll-like receptor signalling pathway”, and “Jak-STAT signalling pathway”, and the number of pathways related to immunity increased over time. The representation of the TPM values of the DEGs belonging to these pathways in heatmaps clearly demonstrated that the number of genes induced by NNV challenge increased substantially over time (**Supplementary Figure S4**).

### 3.7 Expression Analysis of the Main Gene Groups Linked to Innate Immune Response

**Figure 8:**
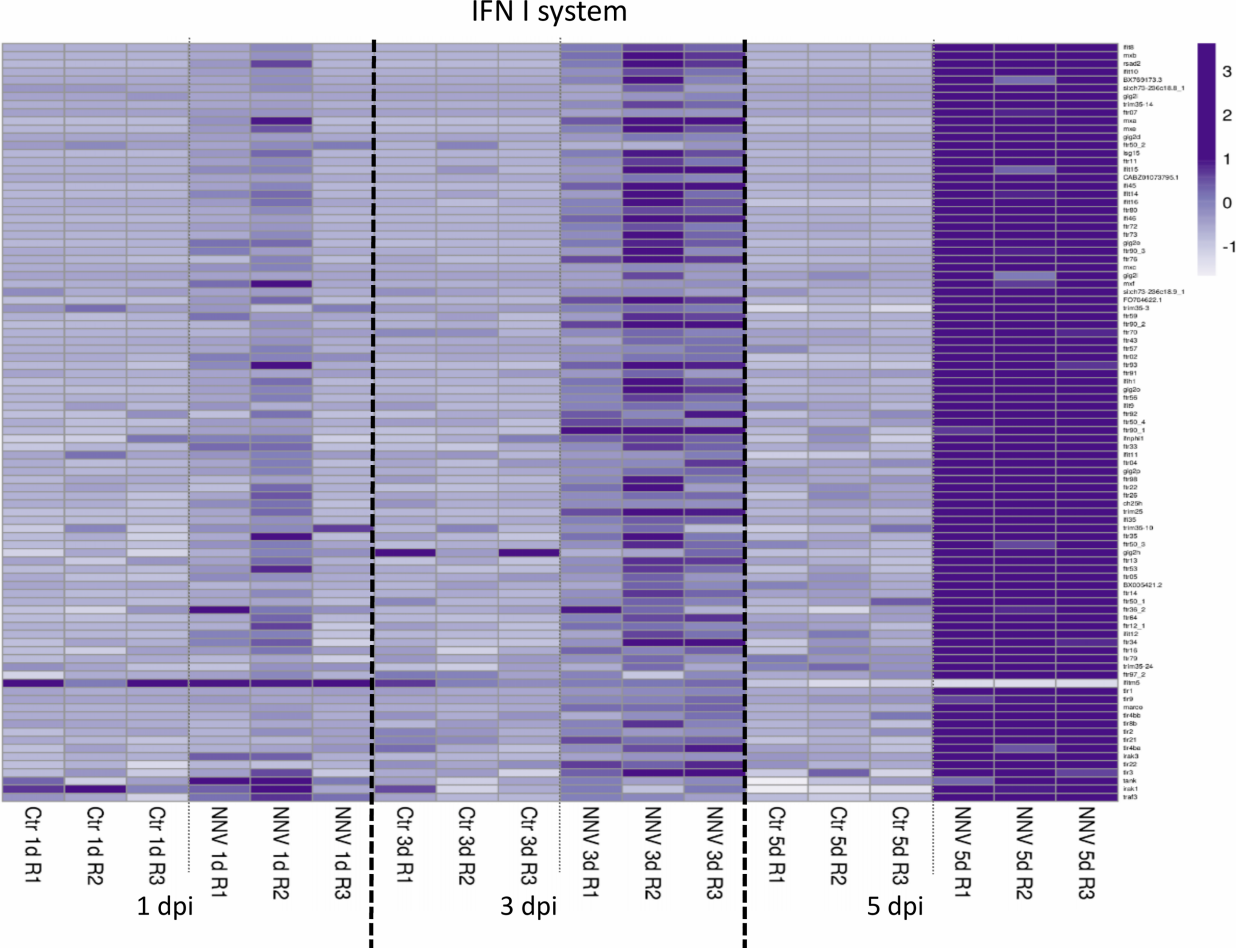
Heatmap representing the DEGs linked to the type I IFN system at 1, 3, and 5 dpi. A heatmap was constructed with the TPM expression values of the DEGs. Expression levels are represented as row-normalized values on a white–purple colour scale.

**Figure 9:**
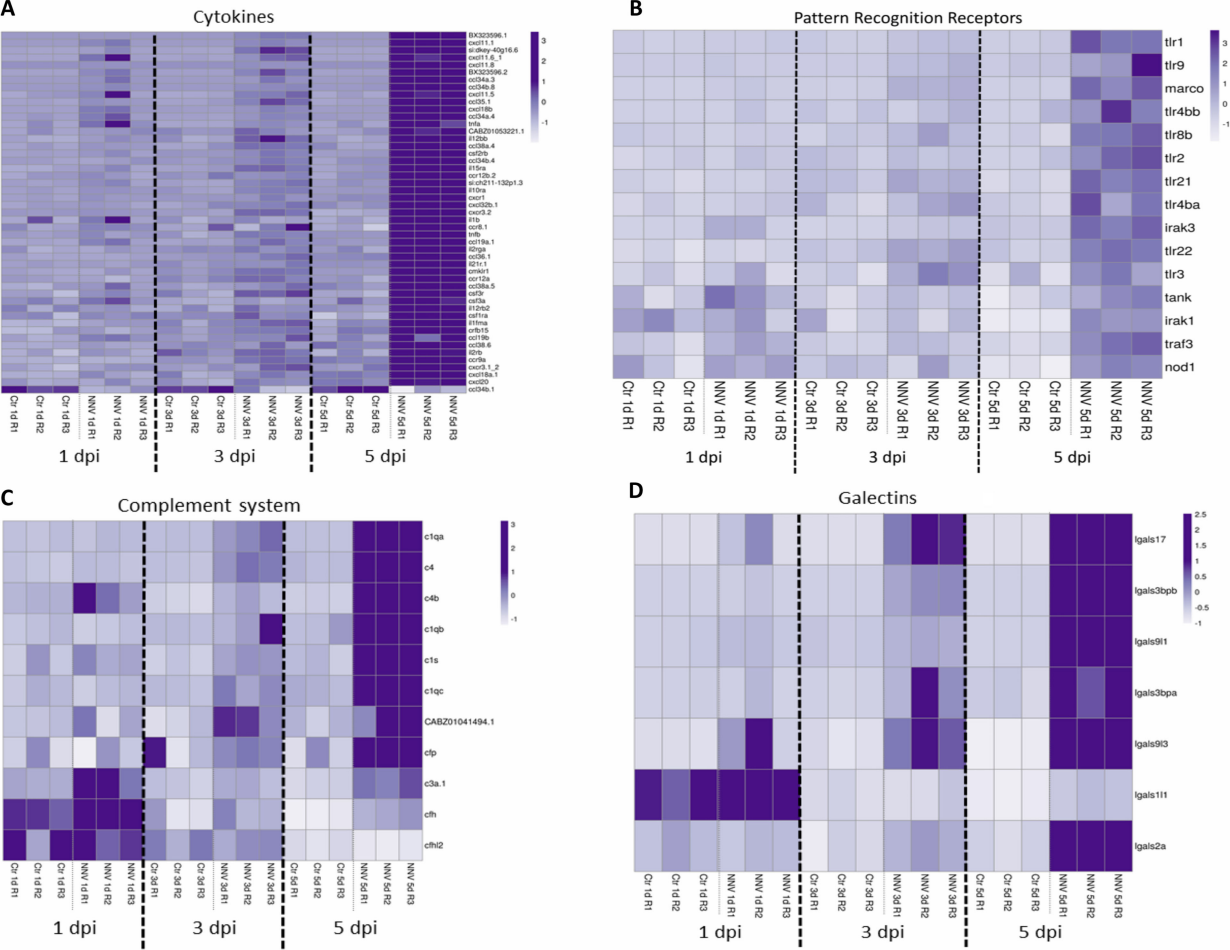
Heatmaps representing the DEGs belonging to different immune categories at 1, 3 and 5 dpi: **(A)** cytokines; **(B)** pattern recognition receptors; **(C)** complement system; and **(D)** galectins. Heatmaps were constructed with the TPM expression values of the DEGs. Expression levels are represented as row-normalized values on a white–purple colour scale.

Different heatmaps were constructed to easily visualize the expression pattern of some of the main groups of immune genes regulated in zebrafish larvae during infection with NNV. As type I IFNs are the main regulators of the antiviral immune response in vertebrates, a heatmap was constructed with those DEGs directly linked to this antiviral mechanism and modulated at least at one of the sampling points by infection with NNV. For this, both interferon regulatory factors (IRFs), the type I IFNs themselves and a multitude of interferon-stimulated genes (ISGs) were considered. As expected, based on the time-increasing replication of NNV, some of these genes were already affected at 1 dpi, but the number of DEGs modulated substantially increased with time as the infection progressed (**Figure 8**). Due to the pivotal role of type I IFNs in the defence against viruses, these cytokines were considered separately from the other types of cytokines (chemokines, interleukins, colony-stimulating factors and tumour necrosis factors). A heatmap representing these other differentially expressed cytokines also reflected a strong overexpression of a multitude of them during the course of infection, with the exception of *chemokine (C-C motif) ligand 34b, duplicate 1* (*ccl34b.1*), which was significantly inhibited at 5 dpi by NNV (**Figure 9A**).

All of these cytokines mentioned above are induced after the recognition of pathogen-associated molecular patterns (PAMPs) by different pattern recognition receptors (PRRs). As mentioned above, the KEGG pathway enrichment analysis showed that two PRR pathways, the “RIG-I-like receptor signalling pathway” and the “Toll-like receptor signalling pathway”, were highly modulated by the virus at the three sampling points, and the “NOD-like receptor signalling pathway” was also enriched at 5 dpi. Therefore, we wanted to analyse in a more detailed way how the different differentially expressed PRRs were affected by the virus. As expected, numerous PRRs (*tlr1*, *tlr2*, *tlr3*, *tlr4ba*, *tlr4bb*, *tlr8b*, *tlr9*, *tlr21*, *tlr22*, *nod1*, and *marco*) and downstream signalling components (*irak1*, *irak3*, *tank*, and *traf3*) were also upregulated in a time-increasing manner (**Figure 9B**). Similar results were observed for different members of the complement system (**Figure 9C**) and the galectin family (**Figure 9D**).

## 4 Discussion

VER disease has an important economic impact on marine aquaculture worldwide, although natural outbreaks have also been detected in several freshwater species (59). While considerable advances in the knowledge of the disease and genetic variability of NNV have been conducted in recent years (59), host–virus interaction mechanisms need to be further studied to help develop effective antiviral strategies.

Zebrafish are a model organism widely used for biomedical research (60) and for studying different concerns linked to fish aquaculture production, such as skeletal malformations (61), pigmentation abnormalities (62), adaptation to prevalent stressful conditions in the aquaculture industry (63), nutrition (64) and infectious diseases (65). It is well known that zebrafish provide significant advantages for understanding host–pathogen interactions in the context of a complete vertebrate. For that reason, zebrafish have been increasingly used for modelling infectious diseases, including those caused by viruses (10), although their use in studying NNV infection remains poorly explored.

Although Furusawa et al. (40) reported that zebrafish were not susceptible to NNV, a year later, Lu et al. (23) were able to reproduce nodavirus infection in this fish species after intraperitoneal injection. They observed a time-increasing viral replication in the brain that peaked at 3 dpi, and histological studies of this tissue revealed lesions similar to those observed for naturally susceptible species (23). Moreover, zebrafish larvae microinjected with NNV showed a high mortality rate at 24 hpi (98%) compared with the mock-injected larvae (24%), although the age of the larvae and route of microinjection were not specified by the authors (23). In that work, the pivotal role of the type I IFN system in protection against NNV was also demonstrated, as the treatment of zebrafish larvae with recombinant IFN conferred protection against viral infection (23). Afterwards, Morick et al. (24) described the infection of zebrafish larvae by bath challenge and the protection conferred by ribavirin treatment (41). However, these works did not fully use all of the advantages that this model species offers to us. In this study, we wanted to propose the use of zebrafish as a model species for this disease and to use techniques that allow us a deeper understanding of what happens during this viral disease.

First, we aimed to determine the most effective route and age of infection for zebrafish larvae to conduct further studies. After the infection of 3-dpf larvae *via* the brain, *via* the duct of Cuvier (DC), intramuscularly (IM) and by bath challenge, we found that the route that offered a significantly lower percentage of survival against NNV was microinjection *via* the brain followed by microinjection IM. However, DC and bath challenges did not cause significant mortality to the larvae compared with the uninfected control larvae. This contrasts with the data published by Morick et al. (24), who found that 4-dpf larvae were highly susceptible to NNV by bath challenge, although the mortality significantly decreased in 6- and 8-dpf larvae. Based on this, we wanted to determine whether the age of the larvae influenced the susceptibility to the virus; therefore, larvae at 14 dpi were infected under the same conditions as the 3-dpf larvae, and no significant mortalities were observed for any of the infection routes. In agreement with Morick et al. (24), older larvae are more resistant to the virus, which could be due to a more developed immune system.

Therefore, 3-4-dpf larvae would be optimal for studying NNV–zebrafish larvae interactions not only for their higher susceptibility but also for their transparency, enabling the use of different imaging techniques. Among them, whole-mount immunofluorescence allowed us to confirm that, as occurs in naturally susceptible species, after IM infection, NNV can migrate to the brain and ocular region. Although different natural routes of infection have been proposed for NNV (epithelial cells, gills, and nasal and oral cavity) (59), the process of viral migration from the muscle to the central nervous system (CNS) was largely confirmed, and due to the higher infective efficiency of the IM route (66), it is the most extensively used route for experimental infection in farmed fish species (32, 34, 38, 67, 68). The spread of infection from the muscle to the CNS may occur through nerve axonal transport (29, 69). As a consequence of the damage caused by NNV in the CNS, one of the main characteristic symptoms of VER disease is the erratic swimming of infected fish. The small size of the zebrafish larvae allows us to better control the experimental conditions *in vivo*, as very large spaces are not required; therefore, certain variables, such as the temperature, are more controlled. When the swimming behavior of 3- and 14-dpf NNV-infected larvae was compared with that of uninfected larvae, changes in velocity, directionality, and Euclidean and accumulated distance were specifically observed in 3-dpf larvae infected *via* the brain, which is probably due to the faster and higher replication of the virus in these larvae.

However, as expected, zebrafish larvae are already able to respond to the virus at early stages of development. The use of zebrafish transgenic lines to analyse the migration of innate immune cell types to the brain after NNV infection through this route revealed a significant migration of neutrophils (Mpx+ cells) and myeloid precursors with lysozyme activity (Lyz+) but not macrophages (Mpeg+). Although the *lyz* gene is expressed in both granulocytes and macrophages, its expression is considerably higher in granulocytes (70), which was also determined in mammals (71). Therefore, the similar migratory trend observed for the Mpx+ and Lyz+ cells is because, in both cases, these cells most likely correspond exclusively to neutrophils. Neutrophils are the first immune cells recruited to sites of infection, where they play important protective functions, including the phagocytosis of infectious agents (72). Nevertheless, although certain viruses have been detected inside neutrophils, it is not clear whether that is a consequence of the active infection and propagation of these specific viruses within neutrophils (72). Neuroinvasion is a rare phenomenon, but certain neuronal viruses, such as NNV, have the ability to colonize the CNS. Among the strategies to reach the brain, a highly protected organ, is the so-called ‘Trojan horse’ strategy, consisting of the camouflage of the pathogen inside immune cells, such as neutrophils and macrophages (72). However, immunofluorescence analysis showed that NNV and Mpx+ cells do not colocalize; consequently, neutrophils do not appear to phagocytose NNV particles as a protection strategy, but they are not used as ‘Trojan horses’ either.

Interestingly, although we did not observe migration of macrophages to the site of injection, the expression of the macrophage marker gene *marco* significantly increased during the course of the infection, reaching higher fold-change values than that observed for the *mpx* gene. It is possible that macrophages migrate to the brain at later infection stages, as cell migration was evaluated until 3 dpi; at this sampling point, the expression of *marco* was not affected by the infection, reaching significant overexpression at 5 and 7 dpi. In contrast, the *mpx* gene showed higher overexpression at 3 dpi. Because both specific markers are already present in 2-dpf larvae (73, 74), these results could suggest a similar pattern to that observed in mammals: resident low immunoreactive macrophages recognize the pathogens and produce neutrophil chemoattractants and, after a rapid influx of neutrophils to the site of infection, they release chemoattracting factors involved in the recruitment of other immune cells, in particular inflammatory macrophages (75). However, more investigation will be needed to elucidate neutrophil–macrophage interactions after NNV infection.

To shed more light on the immune response of zebrafish larvae to NNV, we conducted RNA-Seq analyses of NNV-infected and control larvae at 1, 3 and 5 dpi. As expected, due to the time-increasing replication of the virus, the number of DEGs also increased with time, with most of them being overexpressed in the infected larvae compared with the controls. The GO terms and KEGG pathway enrichment analyses revealed a strong enrichment in immune processes. Among the KEGG pathways, the signalling cascades of the three main types of PRRs (Toll-like, RIG-I-like and NOD-like) were enriched, and a heatmap representing the DEG PRRs and downstream signalling molecules revealed a strong overexpression of a multitude of them at 5 dpi, especially different TLRs. A variety of TLRs were also found to be induced by NNV infection in European sea bass leucocytes (76) and brain DLB-1 cell line (77), although this intense TLR response was not observed in other RNA-Seq studies of fish or fish cells infected with NNV (38, 39, 68, 78–81). Although endosomal TLRs (TLR3, TLR7/8 and TLR9) are typically considered the antiviral TLRs, since they are specialized in the recognition of viral nucleic acids, certain TLRs anchored to the cellular membrane, such as TLR2 and TLR4, are able to recognize viral proteins (82). Ours results suggest the induction of TLRs involved in both nucleic acid and protein recognition. Interestingly, although RIG-I-like receptors have been described as pivotal PRRs in response to NNV infection (42, 43), we did not observe the induction of this type of receptors in our results. 

The interaction of viral PAMPs with different PRRs initiates the recruitment of adapters and the activation of downstream transcription factors that express a multitude of cytokines, including type I IFNs (83). The expression of type I IFNs, the main cytokines orchestrating antiviral defence, mediates their protective effects through the induction of numerous ISGs (84). The relevance of the type I IFN response was well documented in fish after NNV infection, including zebrafish (23, 42–44). However, how the complete repertoire of members of the type I IFN system responds to NNV challenge in zebrafish remains unexplored. As expected, we found a powerful modulation of a multitude of genes involved in this pathway, which indicates a highly efficient response of zebrafish larvae to the virus. Interestingly, contrary to that observed by Chen et al. in the zebrafish cell line ZF4 (42), we did not observe induction in the expression of the different type I IFNs (*ifnphi1*, *ifnphi2*, *ifnphi3*, *ifnphi4*). This fact if probably due to the fast overexpression of these interferons after NNV infection, returning to basal levels at 24 hpi. Nevertheless, the high representation of ISGs highlights the relevance of the type I IFN system. This contrasts with the total absence of a type I IFN response observed in European sea bass brain and head kidney samples at 24 and 72 hpi, which is mainly characterized by activation of the hypothalamic–pituitary–interrenal axis (stress response axis) (38). More investigation will be needed to elucidate whether the high susceptibility of European sea bass to NNV is a consequence of an impaired antiviral response to this virus. Indeed, when the expression of ISG *mx* was analysed in brain samples from European sea bass and in gilthead seabream (*Sparus aurata*), a fish species classically considered resistant to NNV, the induction of *mx* expression was substantially lower in European sea bass than in gilthead seabream (32).

In addition to the type I IFNs, a multitude of other cytokines were induced, reflecting a powerful inflammatory response to NNV, which is probably mediated in the first instance by neutrophils and then by neutrophils and macrophages (75). Indeed, the early migration of neutrophils to the cephalic region of zebrafish larvae after challenge with the virus could be triggered through the production of a multitude of chemokines and the infiltration of more neutrophils and macrophages. Increases in proinflammatory cytokines have also been observed in different organisms infected with NNV, such as Atlantic halibut (85), European sea bass and gilthead seabream (32), turbot (34), zebrafish (24), or different species of grouper (81, 86). Interestingly, the expression of *tumour necrosis factor alpha* (*tnfa*) was previously suggested to be substantially higher in species susceptible to NNV, such as European sea bass, compared with its expression in species resistant to NNV, such as gilthead seabream, which could indicate that this proinflammatory cytokine may be responsible for a large inflammatory reaction in the areas of infection of this virus, such as the brain, retina and spinal cord, producing a neurodegenerative process that the fish cannot overcome (32). However, this result contrasts with the total absence of overexpression of proinflammatory genes observed after an RNA-Seq analysis of brain samples from European sea bass infected with NNV (38), which could be a consequence of different degrees of disease severity or different evolution patterns as a consequence of a multitude of experimental factors (age and size of the animals, virulence of the NNV stock, temperature, etc.).

The complement system has the ability to recognize viruses and virus-infected cells and trigger effector pathways aimed at neutralizing viruses or killing infected cells (87). The strong induction of a multitude of components of the complement system also evidenced the importance of this immune mechanism in the defence against NNV. The overexpression of different complement members after infection with this virus was already observed in brain (38) or liver (88) samples from European sea bass or in leucocytes infected *in vitro* (76). Moreover, increases in the complement haemolytic activity of serum samples from NNV-infected gilthead seabream were observed at 24 hpi, although this activity was not significantly affected in European sea bass (88), which could also contribute to the different susceptibilities to the virus. Indeed, some viruses are able to develop complement evasion strategies (87), and this ability could vary depending on the fish species. However, whether members of the *Nodaviridae* family are able to destabilize the complement response remains to be elucidated.

Finally, the other group of genes standing out from the DEGs in zebrafish larvae were galectins. These β-galactose-binding lectins, which could also be considered PRRs, are expressed in a multitude of cell types and play major roles in defence against pathogens (89). However, little is known about the role of galectins in viral infections, and although they clearly have a function in viral infections, their mode of action is not completely understood (89). Indeed, some investigations have reported antiviral activity of galectins against certain viruses, but other works attributed a proviral role to certain galectins (88). The recurrent overexpression of different galectin genes in different fish species challenged with NNV (67, 68, 76, 90) also evidence a function of these lectins in the response to this virus. Indeed, zebrafish galectin family members showed antiviral activity against the fish virus infectious haematopoietic necrosis virus (IHNV) through direct interaction with the viral glycoprotein, resulting in reduced viral adhesion to the cells (91, 92). Recombinant *Paralichthys olivaceus* Galectin 1 also showed the ability to neutralize lymphocystis disease virus (LCDV) and exert anti-inflammatory activity during infection with LCDV, but the lower expression of proinflammatory genes could be a consequence of lower viral replication in fish treated with the recombinant protein (93). Similar results were observed for the recombinant European sea bass Galectin 1 against NNV, with a significant reduction in the inflammatory response, although the concrete antiviral mechanism mediated by this galectin remains to be elucidated. Moreover, the overexpression of 7 different zebrafish galectin members after NNV observed in this work could indicate nonredundant and complementary roles in the fight against NNV.

While the numerous advantages of zebrafish as a model organism in biomedical and aquaculture research have been extensively noted in recent years, this work reaffirms the foundations of zebrafish larvae to be considered a model of infection, not only for NNV but also for many other viruses. The experimental challenge of zebrafish larvae can reproduce the disease and mimic the course of the infection, as occurs in other NNV hosts. Zebrafish larvae showed susceptibility to this virus, with significant mortalities after intramuscular microinjection or by directly inoculating the virus into the brain. As in the farmed fish species susceptible to NNV, the virus was able to migrate to the brain after an intramuscular infection, and alterations in swimming behavior were observed during infection, suggesting CNS damage. Larvae were also able to mount an efficient antiviral response at both the cellular and humoral levels. Based on these results, different aspects of NNV pathogenesis, immune response and screening of antiviral drugs could be easily studied in zebrafish larvae.

## Data Availability Statement

The datasets presented in this study can be found in online repositories. The names of the repository/repositories and accession number(s) can be found below: http://www.ncbi.nlm.nih.gov/sra, PRJNA799765.

## Ethics Statement

The animal study was reviewed and approved by CSIC National Committee of Bioethics under approval number ES3605702020012020/13/FUN.01/INM06/BNG.

## Author Contributions

RL conducted the main experimental assays. RL and PP analysed the data, prepared the figures and wrote the manuscript. AF performed the RNA-Seq analyses. AF and BN conceived and supervised the study and edited and reviewed the manuscript. All authors contributed to the article and approved the submitted version.

## Funding

Our laboratory is funded by projects PID2020-119532RB-I00 from the Ministerio de Ciencia e Innovación, 0474_BLUEBIOLAB from EU FEDER Programa Interreg España-Portugal and IN607B 2019/01 from Consellería de Economía, Emprego e Industria (GAIN), Xunta de Galicia. RL and PP wish to thank the Axencia Galega de Innovación (GAIN, Xunta de Galicia) for their predoctoral ((IN606A-2017/011) and postdoctoral contracts (IN606B-2018/010), respectively. We acknowledge support of the publication fee by the CSIC Open Access Publication Support Initiative through its Unit of Information Resources for Research (URICI).

## Conflict of Interest

The authors declare that the research was conducted in the absence of any commercial or financial relationships that could be construed as a potential conflict of interest.

## Publisher’s Note

All claims expressed in this article are solely those of the authors and do not necessarily represent those of their affiliated organizations, or those of the publisher, the editors and the reviewers. Any product that may be evaluated in this article, or claim that may be made by its manufacturer, is not guaranteed or endorsed by the publisher.
