# Supplementary material for: Zebrafish as a Vertebrate Model for Studying Nodavirus Infections
Source: Front Immunol. 2022 Mar 24;13:863096. doi: 10.3389/fimmu.2022.863096 (PMC8987509; doi:10.3389/fimmu.2022.863096)
Supplement: Supplementary file 4 [file DataSheet_4.pdf]

Supplementary Material

1 Supplementary Tables

Supplementary Table S1. Primer sequences used in this study.

Primer name	Direction	Primer sequence
il1b	Forward	5'-TTC CCC AAG TGC TGC TTA TT-3'
	Reverse	5'-AAG TTA AAA CCG CTG TGG TCA-3'
tnfa	Forward	5'-ACC AGG CCT TTT CTT CAG GT-3'
	Reverse	5'-GCA TGG CTC ATA AGC ACT TGT T-3'
il6	Forward	5'-GCC AAC TGC AAC ATA CCA AA-3'
	Reverse	5'-ACT GAC AGC ACG CAA AAC TC-3'
ifnphi1	Forward	5'-GAG CAC ATG AAC TCG GTG AA-3'
	Reverse	5'-TGC GTA TCT TGC CAC ACA TT-3'
mxe	Forward	5'-AGT CAC CCA ATG TCA GTG CA-3'
	Reverse	5'-GCT GAG AGA TGT ACT GGT TC-3'
mpx	Forward	5'-TCC AAA GCT ATG TGG GAT GTG A-3'
	Reverse	5'-GTC GTC CGG CAA AAC TGA A-3'
marco	Forward	5'-AAG GAC CCA CAG GAC AAC AG-3'
	Reverse	5'-ATG TGG TGA TGC TCC TCC TC-3'
NNV Capsid protein	Forward	5’-GAC GCG CTT CAA GCA ACT C-3’
	Reverse	5’-CGA ACA CTC CAG CGA CAC AGC A-3’
18s	Forward	5'-ACC ACC CAC AGA ATC GAG AAA-3'
	Reverse	5'-GCC TGC GGC TTA ATT TGA CT-3'

2 Supplementary Figures

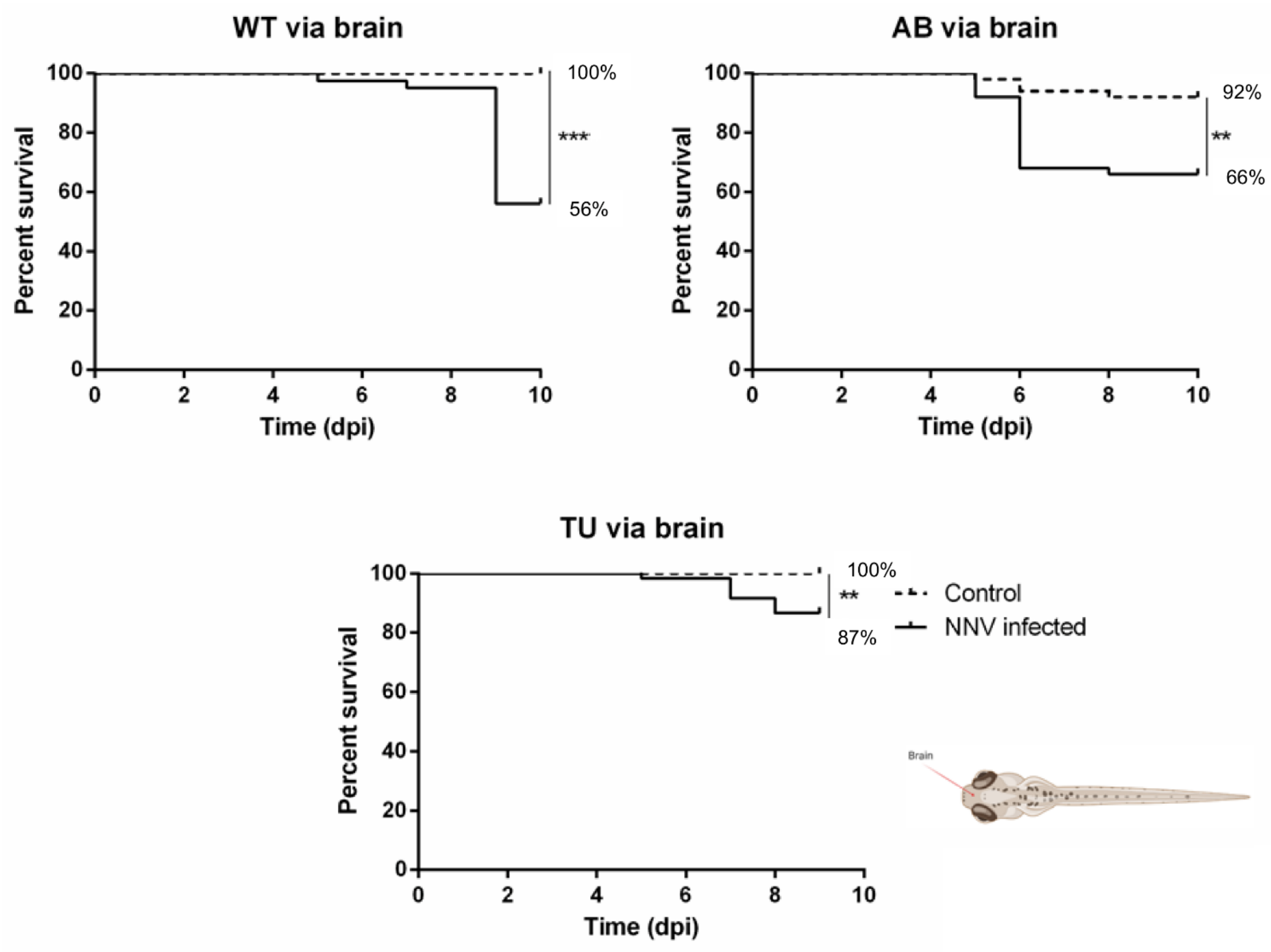

Supplementary Figure S1. Kaplan–Meier survival curves of NNV-infected and uninfected WT, AB and TU zebrafish larvae. Larvae were infected via brain at 3 dpf. Mortality was recorded daily during the next 10 days in the three biological replicates (10 larvae/replicate) obtained for each condition.

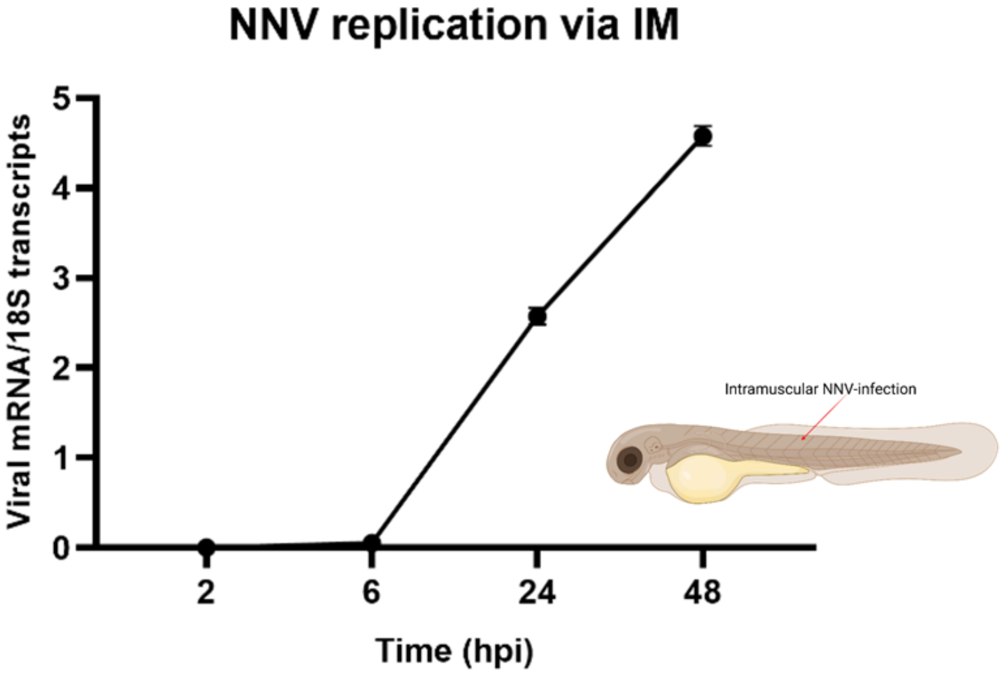

Supplementary Figure S2. Quantification of NNV capsid protein gene expression in 3-dpf larvae infected intramuscularly at different sampling points (2, 6, 24 and 48 hpi) through qPCR. Data are represented as the mean ± SEM of the biological replicates. Statistically significant differences are displayed as follows: ***, 0.0001 > p value > 0.001; **, 0.001 > p value > 0.01; *, 0.01 > p value > 0.05.

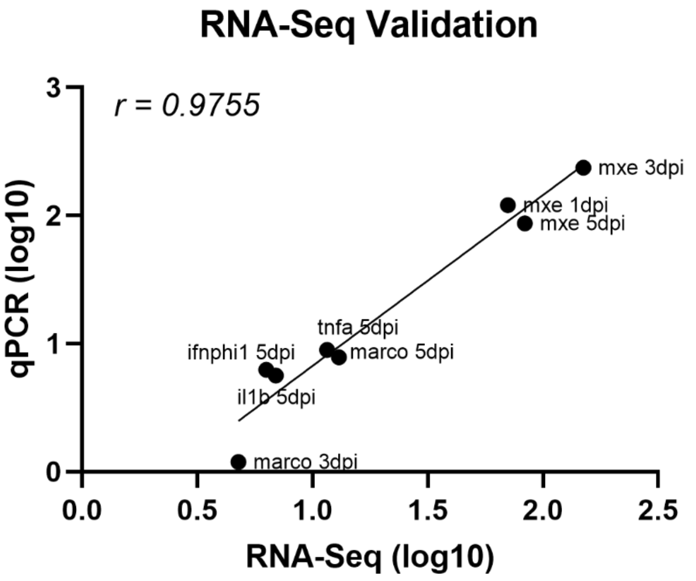

Supplementary Figure S3. qPCR validation of the RNA-Seq results. Correlation between the qPCR and RNA-Seq FC values evaluated using Pearson's correlation coefficient.

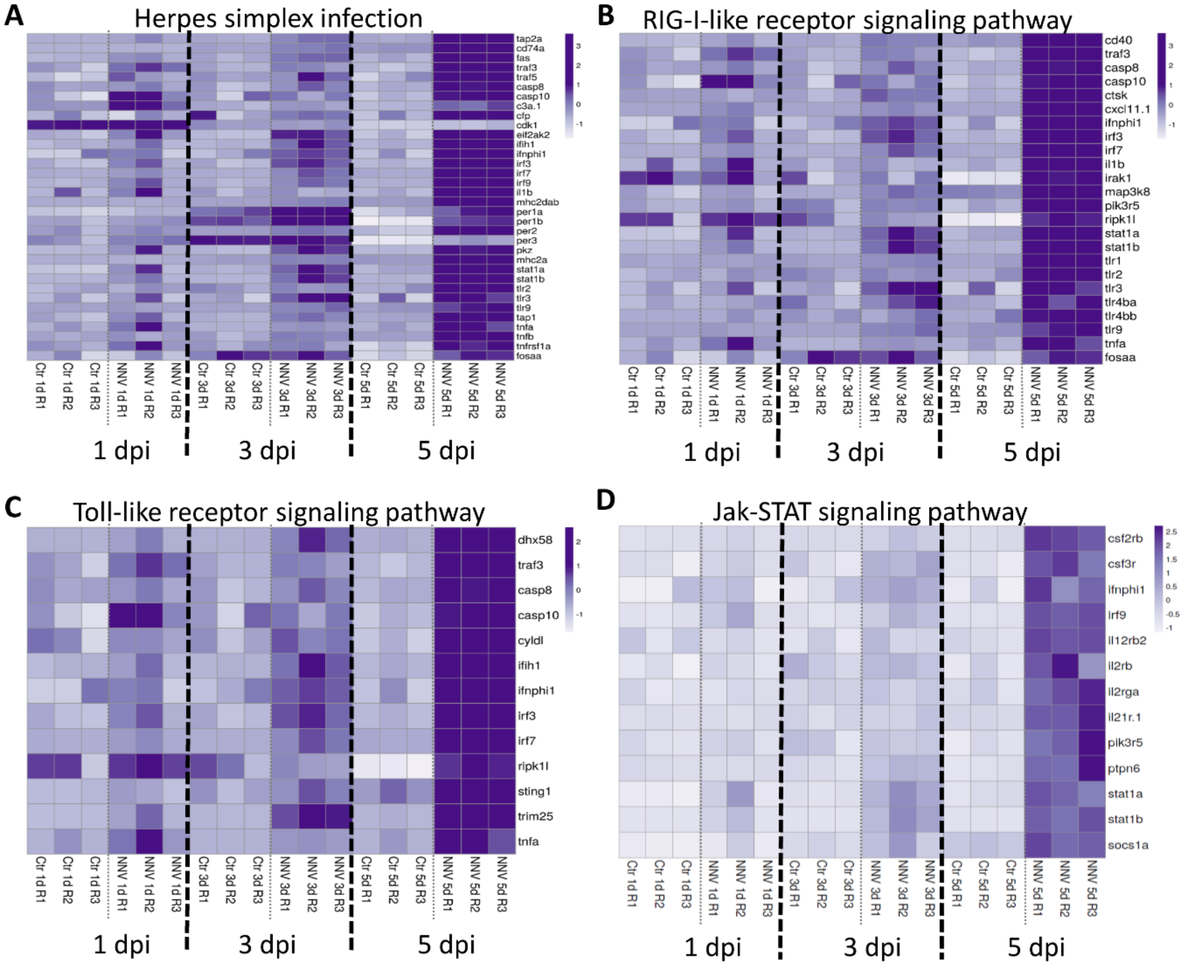

Supplementary Figure S4. Heatmaps representing the log2-transformed TPM expression values of the DEGs related to the four KEGG pathways enriched at the three sampling points: A) herpes simplex infection; **B)** RIG-I-like receptor signalling pathway; **C)** Toll-like receptor signalling pathway; **D)** Jak-STAT signalling pathway.
